# Draft Genome Sequences of 10 Bacterial Strains Isolated from an Abandoned Coal Mine in Southeast Kansas

**DOI:** 10.1128/MRA.01001-19

**Published:** 2019-10-17

**Authors:** Brady Steinbock, Rachel Bechtold, Joseph L. Sevigny, Devin Thomas, W. Kelley Thomas, Anuradha Ghosh

**Affiliations:** a Department of Biology, Pittsburg State University, Pittsburg, Kansas, USA; b Hubbard Center for Genome Studies, University of New Hampshire, Durham, New Hampshire, USA; Indiana University, Bloomington

## Abstract

Here, we report 10 bacterial strains isolated from an abandoned coal mine in southeast Kansas to determine their potential for bioremediation through comparison of the genome sizes and distribution patterns of unique metabolic genes. The selected strains belong to the genera *Arthrobacter*, *Jeotgalibacillus*, *Kocuria*, *Microbacterium*, *Pantoea*, *Rhodococcus*, *Vibrio*, *Brevibacterium*, and *Paenibacillus*.

## ANNOUNCEMENT

For over half a century, sizeable areas of land throughout the Midwest were mined for various underground resources, one of the most notable being coal. Once mined, the lands were left for years. Some areas were partially reclaimed following the Surface Mining Control and Reclamation Act of 1977. The major environmental pollutant that became established in those reclaimed as well as abandoned areas was acid mine drainage (AMD) (1). Pyrite, which is usually found in anaerobic environments, is exposed to atmospheric oxygen and water after coal mines are abandoned, leading to the formation of sulfuric acid and iron. The iron precipitates at the shores and beds of mined land, and sulfuric acid runoff dissolves heavy metals into ground and surface water. Naturally occurring acidophilic bacteria in mined land can increase the oxidation rate of pyrite, while other microbes may offer a solution to the detrimental effects of AMD (2). In the present study, whole-genome sequencing (WGS) analysis provided insight into the presence of genes related to stress, resistance, and metabolism. Thus, application of these isolates is not limited to restoration of AMD sites but also could be exploited for bioremediation of heavy metals and recalcitrant chemicals in contaminated sites.

Soil samples were collected from a depth of 5 cm at five specific sites in the partially reclaimed soil as well as from an AMD site. Up to 60 bacterial isolates were characterized using physiological and biochemical tests (3). Out of these, 10 strains were selected for WGS analysis based on their metabolic diversity. Selected pure cultures were grown overnight in tryptic soy broth (BD Difco, Franklin Lakes, NJ), and whole genomic DNA was isolated with a GenElute bacterial genomic DNA kit (Sigma-Aldrich Corporation, Natick, MA) following the manufacturer’s protocol. Finally, a NanoDrop Lite (Thermo Fisher Scientific, Waltham, MA) analysis was performed on each isolated DNA for quantification and to ensure sample purity.

WGS was performed at the Hubbard Center for Genome Studies (University of New Hampshire, Durham, NH). A paired-end library was constructed using a Nextera DNA library preparation kit (Illumina, San Diego, CA) and sequenced with an Illumina HiSeq 2500 instrument to produce 250-bp paired-end reads. The total numbers of reads for all 10 strains are listed in Table 1. FASTQ files were trimmed for Nextera adapters and low-quality bases using Trimmomatic version 0.32 (4). For read trimming, trailing and leading bases were removed if the quality score was below 3. In addition, the reads were scanned using a 4-base sliding window and trimmed if the average quality dropped below 15. Trimmed sequencing reads were then assembled using the SPAdes pipeline version 3.5 (5) with default settings. QUAST version 4.6.0 (6) was used to assess the contiguity of the assemblies, and coverage statistics were calculated by mapping FASTQ reads to the assembled contigs with the Burrows-Wheeler Aligner MEM algorithm (BWA-MEM) with default settings. The assembled genomes were annotated via the NCBI Prokaryotic Genome Annotation Pipeline (PGAP) (7), while the identity of these strains was determined by performing a BLASTn (8) search on 16S rRNA and translation initiation factor-2 (IF-2) gene sequences. The assembly metrics and annotated features are given in Table 1.

**TABLE 1:** Accession numbers, assembly metrics, and annotated features of the sequenced environmental strains isolated from an abandoned coal mine in southeast Kansas

Bacterial species	Strain	GenBank accession no.	Avg coverage (×)	No. of contigs	Total no. of reads	Genome assembly size (bp)	*N*_50_ value (bp)	G+C content (%)	No. of coding genes	No. of rRNAs	No. of tRNAs	No. of ncRNAs^*a*^
Vibrio vulnificus	S-C3	SMZZ00000000	447	31	11,287,962	5,792,187	849,468	46.67	5,330	24	81	6
[Brevibacterium] frigoritolerans	F-D2	SMZY00000000	1,408	21	12,709,518	4,568,742	1,243,018	40.87	4,411	21	77	5
Microbacterium oleivorans	F-B2	SMZX00000000	577	5	7,893,216	3,092,005	1,655,501	46.55	2,911	5	45	3
Paenibacillus dendritiformis	F-A1	SMZW00000000	340	33	10,817,884	6,777,904	735,989	53.98	5,788	21	80	4
Paenibacillus amylolyticus	S-B4	SMZV00000000	267	21	8,167,626	7,067,323	3,885,192	45.81	6,008	27	80	4
Rhodococcus qingshengii	S-E5	SMZU00000000	431	45	12,222,178	6,609,607	474,786	38.17	6,021	32	115	7
Kocuria rosea	S-A3	SMZT00000000	576	18	9,751,028	3,953,419	565,760	72.67	3,503	5	48	3
Pantoea ananatis	F-C2	SMZS00000000	239	29	6,247,448	4,805,071	405,717	53.64	4,346	13	71	20
*Jeotgalibacillus* sp.	S-D1	SMZR00000000	558	49	9,813,060	3,978,119	790,309	41.33	3,789	18	77	5
Arthrobacter nitrophenolicus	S-A1	SMZQ00000000	479	43	8,918,834	4,614,804	320,201	65.99	4,168	8	50	3

a ncRNAs, noncoding RNAs.

### Data availability.

The draft genome sequences of these environmental strains, as well as the accession numbers for both the assembly and raw reads, are available at DDBJ/ENA/GenBank under the BioProject number PRJNA523266, and the described accession numbers are listed in Table 1.
